# A meta-synthesis of phenomenological studies on experiences related to diabetes in Sweden focusing on learning to live with diabetes

**DOI:** 10.1080/17482631.2022.2132640

**Published:** 2022-11-14

**Authors:** Tomoko Hosono, Ayako Tochikawa

**Affiliations:** a Faculty of Nursing, Japanese Red Cross College of Nursing, Tokyo, Japan; b Faculty of Nursing, Japanese Red Cross Toyota College of Nursing, Aichi, Japan

**Keywords:** Diabetes, phenomenology, meta-synthesis, learning, experience, lifeworld, Sweden, Japan

## Abstract

**Aim:**

We aimed to survey the findings of phenomenological studies built up in the field of diabetes care in Sweden.

**Design:**

This is a meta-synthesis of phenomenological studies on experiences related to diabetes in Sweden focusing on learning to live with diabetes.

**Method:**

We proceeded by reference to the seven phases of Noblit & Hare.

**Results:**

We examined papers from seven phenomenological studies on learning to live with diabetes in Sweden. From the papers, which describe the experience of living with diabetes, three overarching themes were selected—perception and awareness of the body; integration and responsibility; and presence of others who provide support. From those describing the expert’s experience, an overarching theme emerged—encouraging reflection in persons with diabetes. We interpreted each of these themes in phenomenological terms and compared them with theories in Japan. Persons with diabetes take responsibility for coping with a range of things brought on by the illness and learn to live with diabetes. Experts support these persons so they can see their world objectively and critically scrutinize it. By building a model of diabetes care in Japan that references Scandinavian human science, it will be possible to find new approaches that support learning at the existential level.

## 1. Background

Diabetes is a disease that requires a person to make adjustments to their lifestyle as they integrate treatment behaviours into daily living (Japan Academy of Diabetes Education and Nursing, 2008). To provide support, nurses educate patients to enable them to make such lifestyle adjustments.

Based on the global trend of increasing prevalence of diabetes, it is projected that 11.3% of the population will live with diabetes by 2030. Diabetes control, therefore, is a challenge throughout the world (The International Diabetes Federation, 2021). In Japan, the number of people believed to have diabetes has increased year after year, of which those aged 70 years or older represent a particularly high proportion (Ministry of Health, Labour and Welfare, 2019). In Sweden, the number of people with diabetes is also increasing every year, with the average age of these people rising (Swedish National Diabetes Register, 2020). Both countries have undergone similar situations and both entire nations have been taking measures. The age-adjusted comparative prevalence of diabetes in 2021 was 5.0% in Sweden and 6.6% in Japan compared to the global prevalence at 9.8% (The International Diabetes Federation, 2021) suggesting that health policies in both countries may have had a positive effect to some extent. Japan has established a policy that sets goals regarding the prevention of the onset and aggravation of the severity of diabetes mellitus. To achieve this goal, not only public health nurses, but all nurses, play an important role in public health care. In Japan, specialized nurses have also been fostered, but they only work in hospitals under the direction of physicians and have few opportunities to work autonomously. In Sweden, diabetes care has been established in primary care since the 1980s. Human resources specializing in diabetes nursing have been developed. These specialized nurses are entitled to open clinics (Edwall et al., 2008) and they play an important role in diabetes care. We believe that nurses, utilizing their expertise in the community as Swedish nurses do, can contribute to protecting the health of the Japanese people. We thought we could reconsider diabetes nursing in Japan and gain a new perspective by referring to community-based diabetes nursing and care in Sweden.

In Japan, a focus on providing education about diabetes to patients, along with instruction on a regimen to combat the disease, is the most common approach. The Japan Academy of Nursing Science (2011) defines patient education as: “Nursing practice that provides patients and their families the knowledge, skills, and attitudes necessary for the patient to manage their own illness, health, medical care, and lifestyle adjustments with the goal of maintaining an optimal level of health. This education is implemented deliberately, systematically, and continuously for the purpose of changing behaviors and forming necessary attitudes and behaviors.” It is presumed that this definition is based on the Knowledge-Attitude-Behaviour (KAB) model proposed in the 1960s in which changes of human behaviour take place as a progression: from the spread of knowledge to the target; to the formation of attitudes desirable for good health; to a resulting change in habits and behaviour (Hinohara et al., 1998) . With this model as the basis, it can be stated that patient education in Japan is an instructional model for supplying knowledge. As the population in Japan started ageing and chronic disorders such as lifestyle diseases increased from the 1970s, society began looking for ways to cope with the rising costs of healthcare. In the 1980s, the government started to allow patient charges of instruction and guidance to be sent to the health insurance system. This aimed at quickly releasing patients from hospital and having them switch to at-home care, including administering insulin injections at home (Katsuyama & Akutagawa, 2012). Due to these, and other developments in medical policies, the aspect of providing instruction is now included as part of clinical medical treatment. To assist in this effort, basic nursing education was also changed. Considering the necessity of patient education, and to improve the ability of nurses to provide it, government rules were amended in the 1980s with regard to designating schools and training centres for public health nurses, midwives, and nurses, resulting in patient education being reassessed. Instructional skills were clarified as part of the training of nurses, and a focus was brought to bear on teaching those skills (Niiya, 2016). A direction was set to start training nurses early on in patient-directed instructional skills and thereby build nursing expertise in patient education. However, because of the difficulty of establishing instructional content and fostering instructional skills in students (Niiya, 2016) in basic nursing education there was a tendency to focus on teaching how to provide patients with the necessary information on their disease and its treatment (Kawaguchi, 2010). In particular, the time allotted for practical training in chronic illness nursing was very short, and the type of study content that could produce results and be carried out tended to be creating a pamphlet and sharing information from it (Yamamoto et al., 2005). Students who went through this kind of practical training, once in the clinical setting and faced with the task of providing patient education, would rely on the methods they learned in their basic nursing education and tended to simply supply knowledge. So, patient education in Japan has heretofore been based on an instructional model. Plus, in the name of giving instruction as part of medical services paid for by health insurance, nursing education was aimed at the teaching of instructional skills, while patient education was seen from the perspective of “giving instruction” which tended to be the supplying of knowledge.

Additionally, after Japan was defeated in World War II by the USA, the General Headquarters of U.S. Occupation reorganized nursing education (Tsuboi & Sato, 2002), and educated Japan’s nurse leaders in the USA. The leaders received financial support from an American foundation to have the opportunity to study abroad. After returning to Japan, they introduced American nursing methods and theory to Japan (Ryder, 1983). It is clear from this background that much development in this field in Japan can be referenced to North American theory. From basic education we learn Orem’s self-care nursing concepts, which are sometimes also integrated into the nursing process itself (Hickey, 1990). The concept of self-management is also used in chronic nursing. This is based on the self-efficacy theory and its aim is to actively involve patients in their own care (Novak et al., 2013). For these reasons, nurses in Japan provide support by promoting self-efficacy and encouraging behavioural changes. Under the influence of North American nursing practices, Japan’s diabetes specialist nurses (DNSs) today focus on providing health encouragement and support to individual patients that seeks to change behaviour based on taking intentional action and making adjustments on one’s own.

The authors have reviewed the literature accumulated in the diabetes nursing field on the experiences of people with diabetes and the professionals who support them. During this process we discovered a wealth of findings from phenomenological studies on diabetes nursing in Sweden, with those understandings being applied in the clinical setting. In such literature, the expression “learning to live with diabetes” was found to be employed quite commonly, with “learning” as the key concept. We, the Japanese nursing researchers, who primarily provided guidance through patient education aimed at behaviour modification, had a North American perspective that focused on providing nursing support so that patients would start taking intentional action to maintain their health. We had no prior exposure to this perspective on learning, but soon came to see the difference in focus in both the content and results of patient education. We suspected the difference may be attributable to the nursing education in Sweden, and the view of human beings and ways of thinking of the Swedish people. By exploring the philosophical foundations for diabetes nursing, based on research on learning in Sweden, we set out to clarify the similarities and differences between Swedish and Japanese diabetes nursing in hopes of providing useful counsel for Japanese diabetes specialist nurses.

The purpose of the current research was to survey the findings of phenomenological studies built up in the field of diabetes care in Sweden to interpret anew, by synthesizing previous research, the experiences of learning to live with diabetes and the support given for that learning to take place, and to identify and describe the formation of those experiences based on philosophical foundations.

## 2. Design

This is a meta-synthesis study that is “not to identify similarities of research in a particular area but rather, to dig deep under the surface ‘to emerge with the kernel of a new truth’ and increase our understanding” (Paterson et al., 2001; Beck, 2011). Employing the approach of meta-synthesis, we aim in this study to get to the heart of the experience of learning to live with diabetes as well as the experience of providing support in the area of diabetes care in Sweden, and deepen our understanding of the experiences.

## 2.1. Data collection methods

Literature searches were carried out using the following process. The criteria in this research for selecting the subject literature were those articles targeting the experience of living with diabetes in Sweden. Exclusion criteria were those articles discussing paediatric and gestational diabetes or composite disease. Using the medical and nursing literature databases, CINAHL and PubMed, we searched for articles that include all the following terms: phenomenological, diabetes and Sweden and we got 37 of them after excluding duplicate articles (10 July 2022). From the above, 14 that were not studies about Sweden and those unrelated to learning were excluded. A further 16 were excluded applying the exclusion criteria, leaving seven articles as selected subjects.

## 2.2. Analysis methods

**Figure 1.**
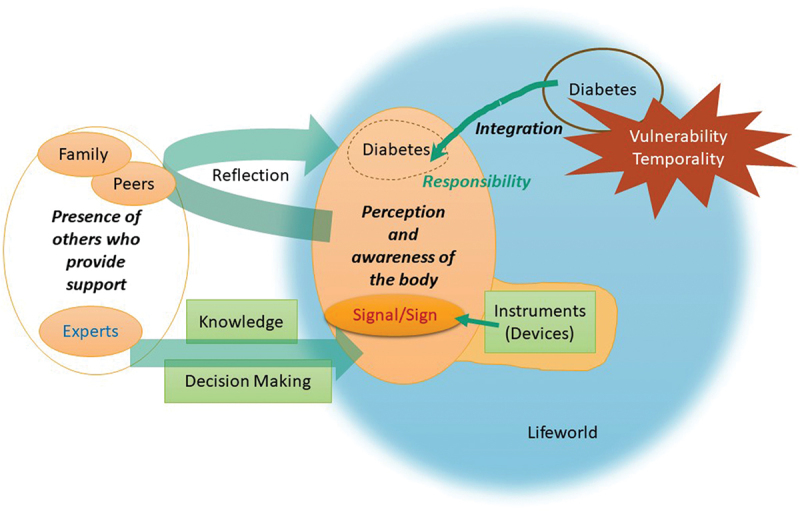
The experience of learning to live with diabetes.

**Figure 2.**
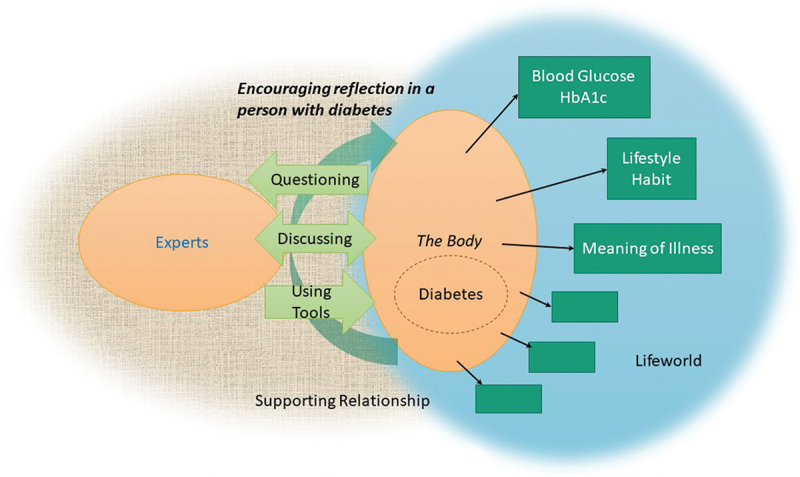
The experience of supporting persons learning to live with diabetes.

We proceeded by reference to the seven phases (in meta-ethnography) of Noblit and Hare (1988). In Phase 1, we identified that our intellectual interest was in the experience of learning to live with diabetes and related support. As this experience of learning to live with diabetes was derived from phenomenological research, in Phase 2, we limited literature to be synthesized to those of phenomenological research. In Phase 3, the selected phenomenological literature was divided by subject into five that describe the experiences of the person living with diabetes and two that describe the experiences of the nurse or other specialists. We read each of them and summarized them in a table (Table I and II). In Phase 4, we identified important metaphors from each article, and the relationships between the articles via the metaphor was hypothesized according to the method of Noblit and Hare (1988) and summarized in tables (Tables III and IV). Having found in these metaphors overarching themes, we re-read the metaphors of each paper. By going back and forth between the metaphors and the themes, we deepened our reciprocal translation. In Phase 5, based on this reciprocal translation, using phenomenological thought and phenomenological research, we further reciprocally translated the overarching themes from the target papers. In Phase 6, we synthesized the translations that made the whole, more than the meanings of the individual parts, and in Phase 7, we expressed the synthesized whole in text and figures (Figures 1 and 2).

**Table I.** Summary of Phenomenological Studies Conducted on Learning to Live with Diabetes in Sweden.

						Methodology				
No.	Year	Auther	Journal	Title	Purpose	quotation of statement	Participant	Data collection	Data analysis	Contens of findings
1	2012	Kneck, Å., Klang, B., & Fagerberg, I.	Journal of Advanced Nursing, 68(11)	Learning to live with diabetes - integrating an illness or objectifying disease	To illuminate the meaning of ‘learning to live with diabetes’ 3 years after being diagnosied	A life world approch (Dahlberg et al., 2008)	13 people who had been diagnosed with diabetes 3 years earlier	Interview	phenomenological-hermaneutic method (Lindseth & Norberg, 2004)	The meaning of ‘learning to live with diabetes’, after 3 year’s experience, is understood as a fluctuation between balance and struggle. Being in balance means diabetes is integrated into the person’s entire existence forming a new way of being. This means that diabetes, body, self and situation are like the links of a chain, holding together and influencing each other and understood as a healthy transition. When balabce is lost, one perspective becomes dominant and a struggle between different perspectives emerges. One could also choose consciously to prioritize perspective, prioritozing what felt most important at the moment, for example, being able to eat as others or feeling good about a correct blood glucose level. This implies that bodily conditions, wn wishes, and circumstances in the life situation or life world(Dahlberg et al. 2008) cannot all be satisfied simultaneously. The learning process of living with diabetes is further understood as a fluctuation between objectifying a disease and intgrating an illness. The objectified disease is separated from oneself and one’s life, whilst the illness is integrated as a subject into the person’s body, self and life world- the lived body (Merleau-Ponty, 1945/2002).
2	2015	Johansson, K., Österberg, S. A., Leksell, J., & Berglund, M.	International journal of qualitative studies on health and well-being, 10	Manoeuvring between anxiety and control: patients’ experience of learning to live with diabetes: alifeworld phenomenological study	To describe what it means to learn to live with diabetes.	Learning to live with diabetes was explored and illumination by the reflective lifeworld research approch (RLR), which is based onphenomenological epistemology, as described by Dahlberg et al. (2008)	12 patients afflicted with type1 or type2 diabetes	interviews	reflective lifeworld research (Dahlberg et al., 2008)	To learn to live with diabetes means realizing that the body no longer functions in a biological way that has been taken for granted. Trusting the body to balance blood sugar levels on its own is no longer possible. Food, activities, and moods influence blood sugar levels in a way that should be mastered, which requires a constant search for knowledge. Learning means taking control and protecting the body from harm in the short- and long-terms. Learning means integrating the disease as change biological body in the understanding of one’s self as a lived body with diabetes as a disease and as an illness. This results in enabling oneself to live to the fullest under new conditions. The learning process demands an understanding that individuals must act on and take responsibility for--a responsibility that is imposed and constantly present. Learning involves handling the changes that occur over time and in available knowledge about the illness and its treatment. Furthermore, learning is driven by the need for safe blood sugar control, the fear of losing such control, and the fear of dependence on checks and future complications. It pertains to the ability to immediately read signals and apply knowledge, with the aim of adjusting blood sugar levels. In so doing, a diabetic controls such levels, thereby fostering the courage to accept the new challenges presented by the illness.
3	2016	Johansson, K., Österberg, S. A., Leksell, J. & Berglund, M.	International Journal of Qualitative Studies on Health and Well-being, 11(1)	Patients’ experiences of support for learning to live with diabetes to promote health and well-being: A lifeworld phenomenological study	To describes the phenomenon of support for learning to live with diabetes to promote health and well-being, from the patient’s perspective.	In this study, the phenomenon of support for learning to live with diabetes is explored and illuminated by the reflective lifeworld research (RLR) approach, based on phenomenological epistemologyas described by Dahlberg et al. (2008)	12 patients living with type 1 or type 2 diabetes	interview	reflective lifeworld research (Dahlberg et al., 2008)	Learning to live with diabetes is supported by self-responsibility, driven by reflection on experiences, curiosity, and a desire to understand and influence one’s daily life and illness processes. Beginning from responsiveness to experience-based feelings in the lived body, reflection supports the ongoing learning process to promote health and well-being. The technology for measuring one’s own blood glucose level is a component of this special support, confirming the body’s feelings and in some cases raising questions that promote the process of reflection. Openness enables an ability of learning support from family and friends, as well as from professional caregivers. Activation of reflection, participation in decision-making, and responsibility are the cornerstones for learning and for a supportive climate. When experiences are explicitly shared with others, progress is made, and lessons are learned from less successful attempts.
4	2016	Kneck, Å., Eriksson, L. E., Lundman, B., & Fagerberg, I.	Journal of Clinical Nursing, 25(19-20)	Encumbered by vulnerability and temporality - the meanings of trigger situations when learning to live with diabetes	To illuminate the meaning of trigger situation experienced in eneryday life when learning to live with diabetes	The study has a life-world approach. A phenomenological hermeneutical method, inspired by the philosophy of Paul Ricouer (1976) and developed by Lindseth and Norberg (2004), was used for interpreting the transcripts of the interviews with person living with diabetes.	13 participants with earlier type I or type II diabetes, over a three-year period after being diagnosised with diabetes	interview	phenomenological-hermaneutic method (Lindseth & Norberg, 2004)	One meaning of trigger situations was an emerged need to try to understand what was happening here and now as well as the future to come. The unpredictability creates insecurity with new unanswered questions and learning needs concerning the body as well as social settings and life itself.Time was an important reference for learning. The past, present and the future had to considered together if a trigger situation was to become a learning situation. By comparing the body in the past with the body at present, an understanding of the body emerged. To consider the future was to be aware probably challenging situations.One meaning of a trigger situation when living with diabetes was a complex relation between learning, control and feeling well. From learning perspective, trigger situations, were an opportunity for learning and understanding more about oneself as well as one’s changed conditions for living. This is described by Tan et al. (2012) as the social support influencing the self-management decisions that person with diabetes take.The meanings of trigger situations were to lose the smooth, unreflected way of meaning an everyday life situation, interlaced with feeling of lost control of how to live with new insights of being vulnerable. Trigger situations meant an opportunity for learning, as well as being demanding, unplanned and with limited freedom of choice. Trigger situations presented life and body as unpredictable.
5	2020	Kjellsdotter, A., Berglund, M., Jebens, E., Kvick, J. & Andersson, S.	International Journal of Qualitative Studies on Health and Well-being, 15 (1)	To take charge of one’s life - group-basededucation for patients with type 2 diabetes inprimary care - a lifeworld approach	To describe patients’ experiences of group-based education using the Taking charge of one’s life with T2D model	In this qualitative study, the phenomenon—patients’experience of group-based education—from the model Taking charge of one’s life with T2D was explored and illuminated using the reflective lifeworld research (RLR) approach, based on phenomenological epistemology	12 patients with T2D the diagnosis should not be older than three years and not less than three months.	groupinterviews and individual interviews	reflective lifeworld research (Dahlberg et al., 2008)	The person changes their understanding and changes, the person changes their understanding of the disease and it becomes a part of their life. The meaning takas place through raised issues and reflections concerning experiences on a concrete and existential level which clarifies and motivates changes. The group contribute to a sense of togetherness and community that inspires continued and active learning. The increased understanding and self-awareness motivate changes in living habits and achievements of own goals. The reflections in the group together with own experiences and reflections that occur between the meetings support the fact that the disease is incorporated with the person’s self-image and life, which constitute learning on an existential level. Learning was supported by a tactful challenging approach.

**Table II.** Summary of Phenomenological Studies Conducted on Supporting Persons Learning to Live with Diabetes in Sweden.

						Methodology				
No.	Year	Auther	Journal	Title	Purpose	quotation of statement	Participant	Data collection	Data analysis	Contens of findings
6	2018	Johansson, K., Österberg, S. A., Leksell, J., & Berglund, M.	British Journal of Nursing, 27(12)	Supporting patients learning tolive with diabetes: A phenomenological study	to explored and illuminated DSNs’ approaches to the subject of supporting the learning of diabetes patients through the reflective lifeworld research approach	This study explored and illuminated DSNs’ approaches to the subject of supporting the learning of diabetes patients through the reflective lifeworld research approach, based on a phenomenological theory as described by Dahlberg et al (2008), which examines the study of the structures of lived experience. ‘Lifeworld’ refers to Husserl’s idea to go ‘to the things themselves’ and describe phenomena as they are lived and experienced by individuals (Husserl, 1973).	3 specialist diabetes nures and 16 nursesworking with a variety of patients with diabetes	interviews	reflective lifeworld research(Dahlberg et al., 2008)	To support patients learning to live with diabetes means the DSN must have a self-critical approach with the insight not to take over the patient’s responsibility for their condition. The DSNs try to support patient learning by starting reflection processes by asking questions with the purpose of clarifying the relationship between, for example, biomedical values and patient actions. The purpose of the support is that the patient should feel responsible for making conscious choices to reduce the risk of diabetic complications. Providing support aims to increase the patient’s willingness and ability to take charge of their condition. Providing emotional and practical support created security by being there for the patient, emphasising the patient’s ability, providing tools and instilling hope and courage.
7	2019	Andersson, S., Berglund, M., Vestman, C., & Kjellsdotter A.	Nursing Open, 6 (2)	Experiences of specially trained personnel of group education for patients with type 2 diabetes—A lifeworld approach	To describe how the group education process for people with type 2 diabetes is experienced by diabetes nurses and dietitians who support the patients’ learning, in a primary care setting.	The phenomenon of this study is diabetes nurses’ and dietitians’ experience of group education of persons with T2D, before, during and after implementation. Using the reflective lifeworld research (RLR), a research approach developed by Dahlberg et al. (2008) based on Giorgi’s (2009) phenomenological approach, the phenomenon is explored and illuminated.	2 registered nurses and 3 dietitians who had special education and long experience of counselling and diabetes care.	Three focus‐group interviews with the nurses and dietitians were conducted before, during and after the group‐based education pro‐gramme,	reflective lifeworld research (Dahlberg et al., 2008)	For health professional, it is challenging to wait for the participants’ reflections, listening to them with an open mind and staying in the background, not deciding the coming subject. The health professionals challenge the participants to think and try to find out the answers themselves not always answering their questions. In a tactful way, they ask the participants to reflect on their own experiences of living with T2D. The didactic model gives the health professionals the ability to meet the unique person in their life with T2D enabling a deep relation to the participants by creating safety, support and trust. A tactful and open approach is required to get all the participants in the group to share their experiences from their daily life with diabetes. Tools such as writing, drawing pictures and selecting amongst other pictures are used to support reflection.

**Table III.** Individual study metaphors of living with diabetes as related to three overarcing themes.

Study	perception and awareness of the body	integrration and responsibility	presence of others who provide support
Kneck et al. (2012)	The body as a basis for decision-making about own health	A flucuation between separating and integrating diabetes into one’s being	Depending on others making decisions to enable contorol of own health
Johansson et al. (2015)	Handling changes in the body	Incorperateing the illness and its treatent into daily life Taking responsibility for acquiring and applying for new knowledge	Serching for new knowledge through others
Johansson et al. (2016)	Technology verifying bodly feelings	Responsibility creating curiousity and willpower	Exchanging experirnces with others
Kneck et al. (2016)	The unpredictable body heightens insecurity with awareness of trigger situations one’s own dependeability		Losing control as being aware of an unchosen dependability on others
Kjellsdotter et al. (2020)	Learning through experiences	Clalyfying your own responsibility	Learning from each other

**Table IV.** Individual study metaphors of supporting a person learning to live with diabetes as related to one overarcing themes.

Study	Encouraging reflection in a person with diabetes
Andersson et al. (2019)	Creating the conditions for all participants to share their experiences Addressing an open question that gives open answers to stimulte the group conversation Not always give the answers to find their own answers Using tools stimurate reflection
Johansson et al. (2018)	Conveying the expert’s berief that the persons could cope with diabetes and were able to instil hope Finding suitable tools that support reflection Supporting to learn by being available for questions, reflection-creaing dialoge and discussions

## 2.3. Ethical considerations

The selected papers were used in conformity with copyrights.

## 3. Results

We examined papers from seven phenomenological studies conducted on learning to live with diabetes in Sweden between 2012 and 2020 (Table I and II). The first study was “Learning to live with diabetes—integrating an illness or objectifying a disease” (Kneck et al., 2012). Later studies were expanded beyond the persons living with diabetes to experts who guided the patients to learn as the subjects. Furthermore, an intervention study was conducted on the person with diabetes group referencing a didactic model on learning among people with a chronic illness—”To take charge in life with long-term illness,” (Berglund, 2011)—and the results published (Andersson et al., 2019; Kjellsdott, 2020). This change of focus reveals a shift from research on learning to live with diabetes to implementation in a process that was becoming systematized.

Below, a synopsis is provided for both subjects for whatever phenomenon was identified in the results of each paper, along with an analysis from a phenomenological standpoint of the underlying elements for each subject.

### 3.1. The experience of the person living with diabetes

#### 3.1.1. Overview of the patient’s experience

“Living with a long-term illness such as diabetes means being challenged by changes in relation to daily life and one’s body” (Charmaz, 1983). The expert needs to understand this kind of lifeworld. Also, the time span “is an essential property of a transition” (Meleis et al., 2000). “More knowledge of the transition of living with diabetes is needed to understand how healthcare staff can facilitate the transition process to help people with diabetes.” With an awareness of this issue, Kneck et al. (2012) began their study on learning to live with diabetes three years after the diagnosis. The person living with diabetes learned “to make decisions by using different sources of information” such as from their body or from other people and learned how to maintain “a delicate balance to create a desired life”. “The meaning of ‘learning to live with diabetes after 3 years’ experience’, is understood as a fluctuation between balance and struggle. Being in balance means diabetes is integrated into the person’s entire existence, forming a new way of being. This means that diabetes, body, self, and situation are like the links of a chain, holding together and influencing each other, and are understood as a healthy transition.”

Kneck et al. (2016) conducted continuous interviews under a longitudinal design over a three-year period after the diagnosis of diabetes “to illuminate the meaning of trigger situations experienced in everyday life when learning to live with diabetes.” Kneck et al. were inspired by the concept of triggers in adult learning of Javis (1987) and defined a trigger situation as “a situation highlighting the discrepancy between earlier experiences and new experiences, as well as a situation to which one has no preset response.” When living with diabetes, a trigger situation had the following meanings. “One meaning of trigger situations was an emerged need to try to understand what was happening here and now as well as the future to come. The unpredictability creates insecurity with new unanswered questions and leaning needs concerning the body as well as social settings and life itself. Time was an important reference for learning. The past, present, and the future had to be considered together if a trigger situation was to become a learning situation. By comparing the body in the past with the body at present, an understanding of the body emerged. To consider the future was to be aware probably challenging situations.” Another meaning of a trigger situation was “a complex relation between learning, control, and feeling well. From a learning perspective, trigger situations were an opportunity for learning and understanding more about oneself as well as one’s changed conditions for living.” Loss of control was also about realizing that they did not choose to rely on others when they experienced situations that they once had been able to handle themselves. Social support influences the self-management decisions that a person with diabetes takes (Tan et al., 2012). “The meanings of trigger situations were to lose the smooth, unreflected way of meaning in an everyday life situation, interlaced with feelings of lost control of how to live with new insights of being vulnerable. Trigger situations meant an opportunity for learning, as well as being demanding, unplanned and with limited freedom of choice. Trigger situations presented life and body as unpredictable.”

Much research has been done in Sweden with regard to learning to live with diabetes. Johansson et al. (2015) explain the need to understand the phenomenon of learning in the context of the lived body of the person with diabetes, conducting research towards that end. “To learn to live with diabetes means realizing that the body no longer functions in a biological way that has been taken for granted. Trusting the body to balance blood sugar levels on its own is no longer possible. It requires a constant search for knowledge to master blood sugar levels. Learning means integrating the disease as change [into the] biological body in the understanding of one’s self as a lived body with diabetes as a disease and as an illness. This results in enabling oneself to live to the fullest under new conditions. The learning process demands an understanding that individuals must act on and take responsibility for—a responsibility that is imposed and constantly present.”

Johansson et al. (2016) studied support for learning to live with diabetes to promote health and well-being, asking the question of how learning to live with diabetes can be facilitated further. “Learning to live with diabetes is supported by self-responsibility, driven by reflection on experiences, curiosity, and a desire to understand and influence one’s daily life and illness processes. Beginning from responsiveness to experience-based feelings in the lived body, reflection supports the ongoing learning process to promote health and well-being. The technology for measuring one’s own blood glucose level is a component of this special support, confirming the body’s feelings and in some cases raising questions that promote the process of reflection. Openness enables an ability of learning support from family and friends, as well as from professional caregivers. Activation of reflection, participation in decision-making, and responsibility are the cornerstones for learning and for a supportive climate.”

Kjellsdotter et al. (2020) clarified how, from the experience of persons living with diabetes, patient learning can be supported by group-based education, based on the didactic model (Berglund, 2011), using the “taking charge of one’s life with T2D” model: “The person changes their understanding of the disease and it becomes a part of their life. The learning takes place through raised issues and reflections concerning experiences on a concrete and existential level which clarifies and motivates changes. The group contribute to a sense of togetherness and community that inspires continued and active learning. The increased understanding and self-awareness motivate changes in living habits and achievements of own goals. The reflections in the group, together with own experiences and reflections that occur between the meetings, support the fact that the disease is incorporated with the person’s self-image and life, which constitute learning on an existential level. Learning was supported by a tactful challenging approach.”

#### 3.1.2. Reciprocal translation from a phenomenological perspective

From each paper we identified important metaphors for the experience of persons with diabetes which we determined were in the relationship of reciprocal translation. Having found in these metaphors three overarching themes: (1) perception and awareness of the body; the body as blood sugar levels, (2) integration and responsibility and (3) presence of others who provide support, we re-read the metaphors of each paper. By going back and forth between the metaphors and themes, we deepened our reciprocal translation. (Table III).

Based on this reciprocal translation, using phenomenological thought and phenomenological research, we further reciprocally translated the three overarching themes related to the experience of learning to live with diabetes.

##### 3.1.2.1. Perception and awareness of the body; the body as blood sugar levels

According to Kneck et al. (2012), the experience of the body had become foundational for decision-making among persons living with diabetes within three years of their diagnosis. When persons with diabetes received signs and signals from their body, they began to become aware of physically having diabetes, and at this time their decision-making became rooted in the body. Physical signs and signals represent symptoms of the illness, but in most cases body awareness was derived from the blood sugar levels taken with a blood glucose metre. The blood sugar levels informed the person who hadn’t noticed any signs or signals of a reaction taking place in their body. Measuring blood sugar was a useful tool in becoming aware of their body. Yet, being controlled by blood sugar levels could make it difficult to rely on physical signs such as hunger. When physical signs and blood sugar levels differed, persons also felt they couldn’t trust their physical sensations.

Johansson et al. (2015) studied the experience of learning to live with diabetes and found that the patient recognized that their body had changed. Improving the ability to recognize that one’s body cannot control its own blood sugar levels, as it was once capable of doing, was identified as one aspect of learning to live with diabetes. Reading the body’s signs, including blood sugar levels, is difficult. Improving one’s ability to be aware of the body is vital for learning to live with diabetes. In order to improve such awareness of the body, various methods used to heighten the person’s awareness are enumerated in the paper, including observation and reflection, and developing curiosity and the will to critically scrutinize one’s lifestyle habits. Raising awareness of the body through blood glucose measurements raises sensitivity to perceiving the blood sugar levels in one’s own body and results in the person coming to understand how those levels came to be. It then becomes necessary to further train sensitivity to understand bodily signals to promote health and well-being (Johansson et al., 2016). It was found that technology used for that purpose, such as blood glucose metres and urine strips, as tools for learning about one’s body enables verification of the connection between knowledge and bodily feelings (Johansson et al., 2015, 2016). In group education, participants cooked different recipes together. After eating each dish they checked out everyone’s blood glucose levels using a device and they became able to see the actual impact on the body (Kjellsdotter et al., 2020). From the time of being recently diagnosed to living long-term with the diabetes, honing one’s sensitivity towards bodily feelings, including to blood sugar levels, is part of learning to live with diabetes. To put it the other way around, unless one hones that sensitivity, it remains difficult to be attuned to the body, which is a situation that calls for learning.

Kneck et al. (2016) clarified that having experienced the unpredictable body as a weakness they became more aware of living as an ever-changing existence, which constitutes triggers that create new understandings of life. The awareness of the unpredictable body caused by diabetes is harsh and unplanned, meaning that only a limited choice of freedom is available, but it is also an opportunity to engender learning towards a new understanding of life. Thus, the involvement of altered awareness of body in learning is significant.

As the person with diabetes gets in the habit of using a blood glucose metre, the device becomes “a bodily auxiliary” (Merleau-Ponty, 1945/2002, p. 176), and “an instrument” (Merleau-Ponty, 1945/2002, p. 176) to help the person perceive their body. When the person becomes able to understand the fluctuation of their blood sugar levels through the use of that instrument, they become able to identify when various blood sugar levels occur. This leads to greater sensitivity in perceiving blood sugar levels and the understanding of one’s own body as revealed through blood sugar levels, which is “a new use of one’s own body” (Merleau-Ponty, 1945/2002, p. 177). This ability to see one’s own body with its diabetes is equivalent to being able to read even slight signs and bodily signals.

##### 3.1.2.2. Integration and responsibility

Kneck et al. (2016) followed lifeworld for three years after a diagnosis of diabetes and clarified that the trigger situation created by sensing life and body as unpredictable propelled learning. Being encumbered by vulnerability and temporality by earlier familiar situations raised the need to understand a changed life and body.

As indicated by Kneck et al. (2012), when the person had involved diabetes in their body and life and formed a new way of being that achieved an integrated balance, diabetes became part of a “new me”. As stated by Johansson et al. (2015), over the long term, those with diabetes are “incorporating the illness and its treatment into daily life,” which is learning. “Being diagnosed with diabetes can be viewed as a catastrophe that is difficult to come to terms with,” but “after some time, the illness was given a place in the interviewees’ lives.” From this change in lifeworld, it can be inferred that learning to live with diabetes had taken place.

Integrating diabetes and its treatment into one’s life also corresponds to “taking responsibility for acquiring and applying new knowledge” (Johansson et al., 2015). “Finally, learning also means knowledge that the ultimate responsibility and choice fall on the person with diabetes themselves.” Additionally, in terms of promoting health and well-being, “Responsibility supports learning as the patients reflect over their experiences and use their knowledge to calculate the risks and benefits of planned actions and to make conscious choices” (Johansson et al., 2016). Reflection takes place on whether or not to take responsibility for a life with illness and its treatment to reduce the risk of complications and future suffering and promote well-being (Johansson et al., 2016).

Also in group education, “although the experience of illness was manifested in sacrifices and negative feelings such as frustration, anger, sadness and compulsion, it became obvious that they are themselves responsible” for their treatment (Kjellsdotter et al., 2020).

Berglund (2014) cites Heidegger’s concept of authentic/inauthentic with regard to taking responsibility amidst the experience of a learning turning point in the person who has a chronic illness. Most people can live life without thinking about their factuality, which Heidegger called “Das Man,” his term for inauthentic living. People can also strive to be themselves and act responsibly, which he called living an authentic existence. Heidegger reasons that authenticity arises from a person realizing through how they live that they cannot escape their own death, while Berglund (2014) reported that a similar situation occurs by becoming ill. From the chronic illness patient interview data she found that “the illness pushed the realization of one’s own facticity and its terms forward” and that “learning is a patient’s awareness of opportunities and possibilities to influence and control their lives and goals.”

According to the research of Johansson et al. (2015), personal responsibility manifests as the act of the person with diabetes acquiring knowledge to understand how their body reacts. It manifests as the person with diabetes realizing that it is “the new me and not anyone else” who must take responsibility, and this is linked inseparably with acquiring knowledge and understanding their own body. Berglund and Källerwald (2012) explain that genuine learning occurs when the sufferers shift from ignoring their situation to letting the illness be a part of their lives. According to Berglund (2014), “the genuine learning exists on what Eriksson (2000) called the “being and caring” level. In Scandinavian care, the pedagogical approach is given precedence because it raises the person’s quality of life and opens avenues to achieve “the good life” (Takenouchi, 2012, p. 462). Well-being (living well) “is handing down a decision to oneself in each case when faced with myriad possibilities, essentially achieved through self-determination as the only course to take” (Takenouchi, 2012, p. 463). In Scandinavia, the individual’s existence is seen as one in which the person makes choices from a number of possibilities aimed at well-being, towards which learning is directed. Based on that view of human beings, genuine learning in the person with diabetes is realizing the facticity of one’s own body and the time remaining in life, which is authentic existence. From that point of departure, aiming for well-being, the attitude of deciding for oneself when directly faced with choices arises from responsibility. From this attitude based on responsibility also emerges the act of acquiring and applying knowledge, which is genuine learning, or learning at the existential level.

##### 3.1.2.3. Presence of others who provide support

Learning among people with diabetes is also supported by the exchange of experiences with others, including specialists, relatives, acquaintances, and peers (Johansson et al., 2015, 2016; Kjellsdotter et al., 2020). According to Kneck et al. (2016), past experiences of having been able to manage on their own will result in not choosing to rely on others in similar situations. When they are in a state of control lost, however, this represents a lack of social support in self-management choices. Trying to manage by themselves without exchanging experiences with others will further deprive the individual of control. However, others often assist making decisions to enable control of their health (Kneck et al., 2012), facilitate acquisition of new knowledge (Johansson et al., 2015), and start a reflection process (Johansson et al., 2016). “Other people’s stories can activate reflection and motivate change by awakening understanding of what has been done, one’s current lifestyle, and its potential future consequences” (Johansson et al., 2016).

In group-based education, people with diabetes learned from each other, sharing their experiences with one another on dealing with the same illness and trying to make changes in their lives. Reflection provided motivation for this process (Kjellsdotter et al., 2020). People diagnosed with diabetes who were cautioned that they needed to take responsibility for their own treatment and lifestyle still left the cautions “as stimuli to which I respond only absent-mindedly or confusedly,” considering their condition “merely latent”. But when the stories they heard through talking with others who had diabetes resonated with them, they made the shift with “the definite taking of a stand” on participating in their treatment as myself with diabetes (Merleau-Ponty, 1945/2002, p. 423). It is presumed that reflection has occurred in them at this time. Also, when the person feels the concern of friends and family members who want to help and healthcare professionals trying to cure or care for them, they make the shift to “the definite taking of a stand,” accepting themselves as a person who has diabetes, not merely a latent condition. One can justifiably presume that reflection has taken place.

### 3.2. The experience of the experts

#### 3.2.1. Overview of the experts’ experience

The study by Johansson et al. (2018) focused on the diabetes specialist nurses (DSNs) who support patients learning to live with diabetes, because the need was felt for nurses to support learning in such patients against the background of the imperative to make the change from a diagnosis-centred perspective to a more person-centred approach in diabetes care. “To support patients learning to live with diabetes means the DSN must have a self-critical approach with the insight not to take over the patient’s responsibility for their condition. The DSNs try to support patient learning by starting reflection processes by asking questions with the purpose of clarifying the relationship between, for example, biomedical values and patient actions. The purpose of the support is that the patient should feel responsible for making conscious choices to reduce the risk of diabetic complications. Providing support aims to increase the patient’s willingness and ability to take charge of their condition. Providing emotional and practical support created security by being there for the patient, emphasizing the patient’s ability, providing tools and instilling hope and courage.”

Considering that an appropriate perspective and questions that lead to reflection are necessary for learning to live with diabetes, Andersson et al. (2019) clarified how specialists (diabetes nurses and dietitians) support learning in people with diabetes in group-based education using the didactic model (Berglund, 2011). “For health professionals, it is challenging to wait for the participants’ reflections, listening to them with an open mind and staying in the background, not deciding the coming subject. The health professionals challenge the participants to think and try to find out the answers themselves not always answering their questions. In a tactful way, they ask the participants to reflect on their own experiences of living with T2D. The didactic model gives the health professionals the ability to meet the unique person in their life with T2D enabling a deep relationship to the participants by creating safety, support and trust. A tactful and open approach is required to get all the participants in the group to share their experiences from their daily life with diabetes. Tools such as writing, drawing pictures and selecting amongst other pictures are used to support reflection.”

#### 3.2.2. Reciprocal translation from a phenomenological viewpoint

From each paper, we found important metaphors that support learning to live with diabetes. We determined that the accounts were directly comparable and identified an overarching theme: (1) encouraging reflection in a person with diabetes.Then we re-read the metaphors of each paper and deepened our reciprocal translation by means of going back and forth between the metaphors and the theme (Table IV).

From these reciprocal translations, with regard to the overarching theme relating to experience of supporting learning to live with diabetes, we carried out further reciprocal translations utilizing phenomenological thought and phenomenological research.

##### 3.2.2.1. Encouraging reflection in a person with diabetes

It was found that experts conveyed their belief that the persons themselves “could cope with diabetes and were able to instill hope” (Johansson et al., 2018) and created the “conditions for all participants to share their experiences” (Andersson et al., 2019) and supported them to learn “by being available for questions, reflection-creating dialogues and discussions” (Johansson et al., 2018). Experts also addressed “an open question that gives open answers to stimulate group conversation” while “not always giving the answer to allow them to find their own answers” (Andersson et al., 2019). Experts found suitable tools such as metaphors, images, and blood glucose summaries to support reflection (Johansson et al., 2018). In a creative attempt to have the persons with diabetes reflect on their experiences the experts also used suitable tools such as drawing, writing, and photos to have them depict their lifeworld (Andersson et al., 2019).

Thus, encouraging a person with diabetes to carry out reflection will enable them to see the lived world being experienced by them. Reflection can reveal various things such as blood glucose levels, HbA1c, lifestyle and living habits, and the significance of the disease that had not previously been consciously thought about. Reflection allows persons with diabetes to see the lifeworld formed by their relationships with time, space, body, and other people. The nurses help them to critically capture the lifeworld (Johansson et al., 2018). As stated by Merleau-Ponty (1945/2002), “Reflection steps back to watch the forms of transcendence fly up like sparks from a fire; it slackens the intentional threads which attach us to the world and thus brings them to our notice”. (p.XV) Encouraging reflection means making it possible for the person with diabetes to see the relationship between their intentions and lifeworld, raising awareness of the non-reflective way of lifeworld they had been immersed in.

The health professionals who encourage reflection by implementing the didactic model meet illness sufferers as unique individuals (Andersson et al., 2019). Getting the individual to lay bare the unique lifeworld built upon their life intentions through the process of reflection is a way for the health professional to help the person become more intimate with their own existence as a person with diabetes. This is because the health professionals attach importance to the context of the lifeworld of the person with diabetes and how they assign meaning to their lives. This kind of attitude helps those with the illness come to trust the health professionals.

### 3.3. The experience of learning to live with diabetes and support for it

Concerning the experience of learning to live with diabetes and that of supporting these people, we synthesized the translations making a whole that is more than the individual parts imply and expressed in text and figures (Figures 1 and 2).

For a person living with diabetes (Figure 1), receiving signs and signals from the body, such as symptoms and blood glucose levels, signified being aware of the body with diabetes. At this point, the body was the basis for decision-making. Signs and signals often came from the blood glucose measuring device, which was an instrument considered to be part of the body.

The starting point of learning to live with diabetes was trigger situations in which one realized that one’s body and life were encumbered by vulnerability and temporality. The diabetes that one had separated from oneself was integrated into the person who became the “new me” and allowed diabetes a place in life.

Others also support one’s learning to live with diabetes. One may rely on others to make decisions and acquire knowledge from others. And others stimulate reflection. As the one who lives with diabetes, the person looked to the past, present, and future and reflected on who they are.

Responsibility stirs up learning. By accepting the responsibility, blood glucose levels and other early symptoms serve as signals and signs, integrating diabetes into the person, and various actions being brought about, such as acquiring knowledge or receiving decision-making support from others, or reflecting through others.

In the experience of experts supporting learning to live with diabetes of a person with diabetes (Figure 2), the focus was on encouraging their reflection. To this end, they built a supporting relationship, created conditions in which persons with diabetes could ask questions and share experiences among them, and utilized tools to facilitate reflection. Critical reflection supported by experts enabled a person with diabetes to recognize their non-reflective lifeworld without habitual reflections.

## 4. Discussion

### 4.1. Japanese theory on interpreting the experience of learning to live with diabetes

#### 4.1.1. Perception and awareness of the body; the body as blood sugar levels

As indicated by Johansson et al. (2015), the inclusion of changed bodily awareness in learning to live with diabetes is consistent with the foundation for persons with diabetes patients employed in Japan in both embodiment care that cultivates embodied intelligence by deepening understanding of the body and rebuilding trust in the body (Nonami et al., 2016) à la Benner and Wrubel (1989) and the care model that works with body sensations in the person with diabetes (Yoneda, 2003). Understanding bodily sensations and the body in terms of blood sugar levels, and becoming able to live life to the fullest as a “new me” with that body, are also consistent with Laesen & Hummel (2013) and associates’ conclusions about the process of adaptation to chronic illness in becoming able to achieve the best results physically, mentally, and socially.

#### 4.1.2. Integration, responsibility and presence of others who provide support

In terms of the experience of people living with diabetes within three years of their diagnosis (Kneck et al., 2012) and the experience of learning to live with diabetes (Johansson et al., 2015, 2016), diabetes is described as leading to finding a new overall balance, expressed as an existential integration. This overlaps with trajectory management (managing living and coping with the illness as it progresses) as described by Strauss (1984) and the concepts of a psychological “coming to terms” and the “reknitting” of the biography (Woog, 1992, p. 18). In the illness trajectory theory, however, the patient is not seen as an “existence”. Also, the experience of wavering in integrating an objective view of the disease with the actual illness derives from the incongruity of objective understanding versus subjective experience, described by Kleinman (1988), Benner and Wrubel (1989), and Toombs (1993). Having a subjective view of the illness in the lived body, distancing the objectified disease, is to experience wavering. This uncertainty stems from the view of humanity as an existence living with the illness.

Taking responsibility closely resembles the concepts of the caring response described by Benner et al., and the “taking charge” described by Ishii (2019). The process of reflection developing from awareness of the body’s signals and numerical values can be understood as adherence—the ability for the person to take in and follow the instructions of the health professional—which also overlaps with a demonstration of checking one’s own condition and assessing it—self-monitoring. Contraposed to an openness that promotes reflection may be “the difficulty of telling to others” à la Kuroe (2011), or stigma.

#### 4.1.3. Encouraging reflection in a person with diabetes

The experience of the nurse is to support patient learning. As reported by Johansson et al. (2018), the DSN does not “take over” responsibility for the patient’s condition, but is present to facilitate the patient’s critical scrutiny of their own illness; this strategy is similar to the andragogy of Knowles (1980), a theory of adult education. An increase in self-determination is a feature of andragogy. Furthermore, this way of thinking connects to Two types of caring by Benner and Wrubel (1989) quoting Heidegger (1927/1962)—not “the kind of solicitude that leaps in and ‘takes over for the Other that with which he is to concern himself’ (pp. 158–159)“ but “the kind of solicitude that ‘leaps ahead’ of the Other, ‘not in order to take away his (or her) ‘care’ but rather to give it back to him or her authentically … .’ (pp. 158–159)“ The nurse takes an approach to get the individual to cope with and take control of their own diabetic condition. One form of support for learning how to take a self-critical approach is encouraging reflection (Johansson et al., 2018). The DSNs ask questions to get the patient to reflect upon the relationship between biomedical values and their actions. “The purpose of the support is that the patient should feel responsible for making conscious choices to reduce the risk of diabetic complications” (Johansson et al., 2018).

Andersson et al. (2019) reports that in the experience of supporting learning in group education, it is challenging for the health professional to wait for the participants’ reflections. Although not in group education, a nursing practice single case study in Japan conducted over eight years reports on “watchful waiting nursing” with a patient whose diabetic nephropathy had progressed and who would “keep working to the last moment” to avoid dialysis; nurses provided support by going along with the patient’s wishes and waiting for a suitable opportunity to introduce dialysis (Higashi, 2018). While waiting, the nurses created opportunities for reflection in the patient who was worried about their worsening renal functioning. How that approach supported the patient’s decision-making on starting dialysis was also analysed, and the results suggest that the “waiting” attitude of the care provider imparts a sense of security that allows the patient to reflect, and may provide encouragement. In Japan a large part of the literature on group education is still practical reports, so the issues going forward are conceptualization and model creation.

### 4.2. Possibilities for diabetes care in Japan suggested by learning to live with diabetes in Sweden

The Japanese Nursing Association, which plays a central role in Japan, defines patient education as “nursing practice that provides education in terms of knowledge, techniques, and attitude”. Textbooks have been created for basic training based on this definition, and nursing students learn to provide educational content unilaterally as patient education (nearly equal to patient instruction). Additionally, the vast majority of qualified specialists in diabetes care working on the medical front in Japan are certified diabetes educators (CDEJs) directed by doctors, and as the name of their qualification indicates, their stance is grounded in unilaterally providing educational content—instruction. In addition, in Japan’s medical system, to strengthen medical instruction given individually to patients with diabetic early incipient nephropathy and a more advanced condition, a mandate was put into effect in 2012 for inclusion in health insurance remuneration for medical expenses of a “charge for medical instruction to prevent dialysis in diabetes.” Another inclusion is a “charge for medical instruction in at-home self-injections” that is paid when medical instruction is given to patients who must administer an injection by themselves at home or at a geriatric facility. The term “instruction” is thus used regularly within the medical system. Since it has become a general-purpose word in Japan in the medical field, even if unintentionally, nurses and CDEJs take an instructional stance in practicing patient education.

Yet, certified nurse specialists in chronic care nursing who study diabetes nursing as a specialization, as well as nurses certified in diabetes nursing, do not take an instructional approach. Rather, they study and attempt to apply in practice a patient-centred theory such as self-efficacy or andragogy that encourages patients to participate in their own treatment. The problem is that the number of such specialist nurses is low in comparison to the total number of patients in the country. And, much as self-efficacy is the theoretical basis for behaviour therapy, according to Bandura (1977), it is a stance of behaviourism and cognitivism. This thinking is grounded in Descartes’ mind-body dualism, which separates observable physical behaviour from subjective mental activity in the doer. This cognitivist view of the human being inevitably regards the mind as the unitary producer of meaning, and sees behaviour as a result of thought (the “thinking-makes-it-so” idea) (Benner & Wrubel, 1989, pp. 35–38). In the context of diabetes nursing, the patients’ diet, activities, and other actions with regard to medical treatment are taken because “thinking makes it so.” For this reason, support is provided so that the patient with diabetes can have efficacy expectations about being able to manage their lives, reach performance accomplishments, or have vicarious experience, etc., raising self-efficacy and aiming for appropriate behaviour modification. In other words, the thinking and practice of Japan’s diabetic nursing leaders are rooted in a view of the human being based on mind-body dualism—getting the patient to change the way they think to effectuate behaviour modification. Also, most general nurses practice educational care from an instructional stance. It is self-evident that in Japan, nursing involvement is aimed at behaviour modification by instructing/educating the patient in terms of individual abilities and attitudes. Recently, in diabetes care field in Japan as well, phenomenological nursing studies that stand on the perspective of persons with diabetes, rather than mind-body dualism, have been reported (Hosono, 2019a, 2019b, 2021; Tochikawa, 2009, 2017, 2020). In the field of diabetes nursing in Japan, the foundation for developing theory and practice based on the phenomenological view of humans has begun to form.

In contrast, in Sweden, around 1980, a transformation took place in university education inspired by human science, since which time the emphasis shifted to American nursing theory, such as the anthropological foundation of Leininger, or the phenomenological foundation of Benner or Watson (Dahlberg, 2018). Since 1990, there has been a progression to nursing studies based on the work of Finland’s Katie Eriksson, grounded in philosophy and pedagogy, Norway’s Kari Martinsen, based on psychology and philosophy, and Sweden’s Karin Dahlberg and associates that incorporates the perspective of a phenomenological lifeworld—an advancement of independent nursing as “caring science” that regards relationships and dialogue as essential (Arman et al., 2015; Dahlberg, 2018). The Scandinavian human science has given hugely important influence on the development of research, education and clinical practice in the region since the 1990s to date (Arman et al., 2015). In caring science as human science, quite apart from scientific evidence, the age-old values or meanings that accompany people’s lives are recognized and taken as ontological evidence that has academic value (Arman et al., 2015). Within such a theoretical framework, the diagnosis is secondary; within the context of the illness centred on the patient, primary importance is given to the view of the human being as a whole and the patient as one who is suffering. The fragility of existence is universal; caring arises naturally to alleviate the suffering it involves, and the basis of caring is love. This view of the human being differs from the view of the patient as an individual seeking control over their life independent of their situation when considering self-efficacy and self-care in patients. Weak beings live together in a community where they love others and are loved by others. Caring arises when a person falls ill in such a community, which is recognized as an academic basis.

Amidst such academic advancement, Berglund (2014) constructed a didactic model that is based on understanding the patient as a person living with a chronic disease who becomes changed by the illness and learns to live more authentically, and promotes such learning. “The didactic model is called: The challenge to take charge of life with a long-term illness.” Additionally, chronic phase care based on this model is practiced on patients, bringing together human science theory and practice in the development of caring science (Berglund, 2014; Kjellsdotter et al., 2020). Furthermore, as seen in the prior research discussed in this paper (Andersson et al., 2019; Johansson et al., 2015, 2016, 2018; Kjellsdotter et al., 2020; Kneck et al., 2016; Kneck et al., 2012), diabetes care has also made progress as caring science. Such research has shown that also in the person living with diabetes, as they live with a changed body, they integrate the changes into a new self, creating a place for the illness in their lives; they take responsibility for coping with a range of things brought on by the illness and also experience learning to live with diabetes. Nurses support patients so that they may become able to see their world objectively—the world and way of living they experience in their own time and space, through their body and relationships with others—support that helps them become able to critically scrutinize that world and way of living. By building a model of diabetes care in Japan that references Scandinavian human science in this way, it will be possible to find new approaches to diabetes nursing in Japan that support learning at the existential level rather than the instructional approach that has been taken to date.

## 5. Research limitations

This research was limited to phenomenological research that clarifies diabetes in Sweden and the experience of learning to live with diabetes. Only papers written in English were studied, so similar research results not translated into English could not be examined. The experience of people learning to live with diabetes revealed in literature that does not belong to phenomenological research was not studied.
